# Viscoelastic and biological performance of low-modulus, reactive calcium phosphate-filled, degradable, polymeric bone adhesives

**DOI:** 10.1016/j.actbio.2011.08.008

**Published:** 2012-01

**Authors:** Ensanya A. Abou Neel, Vehid Salih, Peter A. Revell, Anne M. Young

**Affiliations:** UCL, Eastman Dental Institute, 256 Gray’s Inn Road, London WC1X 8LD, UK

**Keywords:** Bone adhesive, Viscoelasticity, Creep, In vitro cell compatibility

## Abstract

The aim of this study was to investigate the effect of reactive mono- and tricalcium phosphate addition on the mechanical, surface free energy, degradation and cell compatibility properties of poly(lactide-co-propylene glycol-co-lactide) dimethacrylate (PPGLDMA) thin films. Dry composites containing up to 70 wt.% filler were in a flexible rubber state at body temperature. Filler addition increased the initial strength and Young’s modulus and reduced the elastic and permanent deformation under load. The polymer had high polar surface free energy, which might enable greater spread upon bone. This was significantly reduced by filler addition but not by water immersion for 7 days. The samples exhibited reduced water sorption and associated bulk degradation when compared with previous work with thicker samples. Their cell compatibility was also improved. Filler raised water sorption and degradation but improved cell proliferation. The materials are promising bone adhesive candidates for low-load-bearing areas.

## 1 Introduction

The ongoing aim of the following research is to develop degradable cements/adhesives that could improve bone repair procedures. With the increasing average age of the population and prevalence of osteoporosis, bone fracture incidence is rising. Fixation alleviates pain after fracture, and through enabling earlier mobility enhances natural bone repair [1]. Classical methods of fixation include the use of metallic plates, pins and screws, but site preparation and device removal cause significant tissue trauma [2]. Furthermore, stress shielding (and associated surrounding bone resorption) may occur as a result of modulus mismatch between rigid materials and lower modulus bone [3]. Moreover, screws and pins have poor anchorage within brittle osteoporotic bone [4].

Device removal concerns have been partially addressed by the development of degradable polylactide-based screws [5]. Injectable degradable flexible bone cement and adhesives, however, could rubber-toughen brittle bone and aid early screw anchorage. Moreover, if sufficiently strong they could, in lower load bearing applications, replace the screw thereby reducing site preparation requirements and hence damage [6].

Polymethylmethacrylate (PMMA) is the most commonly used bone cement [7]. High cement viscosity, modulus, heat generation and shrinkage during setting, combined with lack of chemical interaction, however, inhibit good bonding with bone [8] or metal [9]. The Young’s modulus of PMMA has been reduced through the addition of flexible polymeric powders with lower glass transition temperatures [10]. Calcium phosphate filler addition can also improve PMMA osteoconduction [11]. The mechanical deterioration of PMMA with time, however, causes long-term problems. This may be due to various reasons including wear and effects of biological factors [12]. Furthermore, because it does not undergo controlled degradation, PMMA is poorly suited for use with screws which ultimately require removal. The following study investigates rubber-modified, degradable and potentially more adhesive lactide/methacrylate alternatives.

The specific base polymer, poly(lactide-co-propylene glycol-co-lactide) dimethacrylate (PPGLDMA), selected for this study starts as a triblock ABA oligomer with methacrylate groups at each end of the short polymer chain. It has polylactide A block, polypropylene glycol B block and total molecular weights (calculated from NMR) of 288, 425 and 1171 g mol^−1^, respectively. By keeping the B/A molecular weight ratio relatively high, initial fluidity is achieved [13]. The methacrylate groups enable a rapid crosslinking setting reaction but lactide segments ensure subsequent degradability. The total molecular weight is 10 times higher than that of methyl methacrylate. Heat generation and shrinkage are proportional and inversely proportional to the number of methacrylate per molecule and molecular weight, respectively. Heat and shrinkage are therefore 5-fold lower for the oligomer compared with methyl methacrylate. This should improve bonding ability with bone [8,9]. The flexible polypropylene glycol segment length is, however, short in comparison with much previous work (425–2000 g mol^−1^) [13]. This ensures that the polymerization setting rate [13], material modulus [14] and subsequent degradation kinetics [13] are not overly reduced.

Upon PPGLDMA degradation, acidic groups on large insoluble polymers are initially produced that should temporarily provide a chemical mechanism for cement interaction with bone and metal. Ultimately, water-soluble lactic acid, short-chain polymethacrylic acid and polypropylene glycol are the expected breakdown products that may then be eliminated by the body [13]. At high levels, however, acids may inhibit cell growth and proliferation [15]. Previous qualitative studies suggest that addition of reactive mono- and tricalcium phosphate filler particles (monocalcium phosphate monohydrate/β-tricalcium phosphate; MCPM/βTCP) to PPGLDMA may enhance cell proliferation without detrimentally reducing mass loss kinetics. One aim of this study is to confirm and further quantify this finding, but in addition to address the importance of adhesive sample thickness and preincubation in medium.

Major advantages of raising any calcium phosphate filler content in PPGLDMA adhesives include further reduction in heat and shrinkage during polymerization and greater potential bone interaction. Such fillers upon dissolution also provide components that, rather than requiring elimination, aid bone remineralization. The disadvantages of adding filler could include an increase in modulus. Moreover, these fillers increase fluid viscosity, which could reduce methacrylate adhesive spreading and its ability to adhere to rough surfaces via micromechanical attachment [16]. Basic βTCP alone could buffer acidic products, but being of low aqueous solubility might also slow PPGLDMA degradation [15]. Acidic MCPM at a PPGLDMA surface dissolves more quickly than βTCP and simultaneously catalyses surface polymer degradation [13,17,18]. With combined fillers, MCPM in the bulk of a PPGLDMA polymer reacts with βTCP to form neutral and less soluble dicalcium phosphates. Mixed reactive fillers could therefore help control bulk vs. surface degradation and thereby result in changes in mechanical properties with time [19].

Mechanical studies of PPGLDMA formulations to date have been limited to non-destructive dynamic mechanical analysis [14] and compressive modulus determination [17,18]. Bone cement/adhesives are subject to both static and oscillating load during use. The nature of their response could enhance surrounding bone growth or its resorption [20,21]. Furthermore, in common with other viscoelastic polymeric materials, they may undergo substantial creep behaviour [22], which could limit their application. In this study, therefore, static, dynamic and creep mechanical properties of filled and unfilled PPGLDMA materials are examined.

A further important property of PPGLDMA cements not previously quantified is their surface free energy/hydrophilicity. Overly hydrophilic, low-viscosity bone adhesives might spread too thinly or disperse in surrounding fluids before they can set. After setting they may also absorb high levels of water, potentially causing a substantial decline in strength [23]. Surface free energy/hydrophilicity can also have a major impact upon protein adsorption and subsequent cellular interaction [24,25]. Ultimately, the aim of this study was, therefore, to quantify water sorption, degradation, mechanical properties, surface free energy and mesenchymal stem cell compatibility of both unfilled and MCPM/β-TCP (50 up to 70 wt.%) filled PPGLDMA. Studies have focused on thin films suited to adhesive applications whilst most previous work has investigated thicker discs which are more relevant for bone-filling applications [17–19].

## 2 Materials and methods

### 2.1 PPGLDMA/reactive filler composite preparation

A PPGLDMA consisting of seven monomer units in the propylene glycol block and four d,l-lactide units and one methacrylate group each end, was prepared as previously described [13]. Briefly, this molecule was produced using polypropylene glycol (PPG) of 425 g mol^−1^ average molecular weight as an initiator for ring-opening polymerization of lactide. The initiator to lactide molar ratio was 4. Methacrylate groups were then added using methacryloyl chloride with triethylamine. The predicted resultant average oligomer chemical structure was confirmed by nuclear magnetic resonance (NMR) to be:where R′ is C(CH_3_), *n *= 4, R is –[C(CH_3_) CH_2_O–]_7_.

To improve cell compatibility and monomer purification, the resultant product was dissolved in dichloromethane and sequentially washed with acetone, 1% HCl, 0.1 mol NaHCO_3_, and deionized water [17,18]. The percentage methacrylation efficiency, $E_{f}\text{,}$ was estimated using NMR, assuming seven units of propylene glycol in each molecule and the following equation, to be 91%:

$$\%E_{f}=\frac{{\text{PPG/MA}}_{\text{obt}}}{{\text{PPG/MA}}_{\exp}}\times 100$$

${\text{PPG/MA}}_{\text{obt}}$ and ${\text{PPG/MA}}_{\exp}$ are the obtained and expected propylene glycol/methacrylate ratio, respectively. This was comparable with previous studies [18]. At this level nearly all molecules are likely to have at least one methacrylate group and over 80% have two.

To enable photopolymerization, 10 wt.% hydroxyethyl-methacrylate (HEMA) (Sigma–Aldrich) containing 10 wt.% each of camphorquinone (CQ) and *N*,*N*-dimethyl-*p*-toluidine (DMPT) (Sigma–Aldrich) was added to the PPGLDMA. Equimolar ratios of MCPM and β-TCP at 50, 60 or 70 wt.% was used to prepare composite samples. For preparing polymer and composite films of 300 ± 5% μm thickness (as confirmed using a micrometre), the mixed components were compressed between two acetate sheets. The specimens were subsequently polymerized using a visible light box (Dentsply Trubyte Triad® 2000TM), using a light exposure time of 15 min on each side [13].

### 2.2 Methods of characterization

In the following investigations, formulations containing 0, 50, 60 and 70 wt.% filler were all studied and sample number (*n*) was 3 unless otherwise stated.

#### 2.2.1 Water sorption and degradation in growth medium

Discs of 10 mm diameter and 300 ± 5% μm thickness were punched out of the original film and then placed in separate Sterilin tubes each containing 5 ml of standard growth medium. At 1, 5, 7 and 10 days, discs were removed, blot dried with tissue, weighed and then placed in fresh medium. Medium temperature was maintained at 37 °C throughout. The per cent change in mass was calculated using the following equation:

$$M_{c}(\%)=\left(\frac{M_{t}-M_{0}}{M_{0}}\right)\times 100\text{,}$$

where $M_{t}$ and $M_{0}$ are the wet mass of the specimen at time *t* and initially, respectively.

Early water sorption and subsequent surface mass loss rate were estimated, as in previous work [15] from the intercept and gradient, respectively, of percentage mass change vs. time.

#### 2.2.2 Tensile mechanical characterization

All mechanical testing was undertaken using a dynamic mechanical analyzer (Perkin-Elmer DMA-7e, USA) with unsterilized rods of 2 × 8 mm cut from 300 ± 5% μm thick films. The dimensions of each sample were measured prior to the test with callipers. These measured sample dimensions were subsequently employed in calculations undertaken using Pyris™ software. Tensile mode was used in all the following three test methods.

##### 2.2.2.1 Dynamic mechanical thermal analysis (DMTA)

A dynamic strain of 0.2% at 1 Hz frequency was applied [26,27]. A static tensile force of 110% of dynamic force was applied (*n *= 3) to maintain this dynamic strain. The static force was applied at a value 1.1 times greater than the cyclic load [26,27]. This test was carried out (*n *= 3) in a nitrogen atmosphere to limit any possible polymer oxidation/chemical degradation at higher temperatures. The temperature was raised from −20 to 100 °C using a heating rate of 4 °C min^−1^ [28,29]. The storage modulus ($E^{\prime }$), loss modulus ($E^{\prime \prime }$) and mechanical damping (tan *δ*) were then calculated as a function of temperature using Pyris™ software. Glass transition temperature (*T_g_*) was determined assuming this occurs when $E^{\prime \prime }$ reaches its peak value [30].

##### 2.2.2.2 Quasi-static test

In quasi-static tests, specimens (*n *= 5) of all formulations were tested until failure at room temperature using an initial applied load of 1 mN and loading rate of 100 mN min^−1^ [26]. This test was additionally conducted on the polymer and 70 wt.% filled composite at 37 °C or at room temperature after 24 h in water. The Young’s modulus was calculated from the slope of the initial linear portion of the stress–strain curve, while the ultimate stress was calculated from the final stress before break using Pyris™ software.

##### 2.2.2.3 Creep/recovery experiment

In creep experiments, studies were conducted on the polymer and 70 wt.% filler composite dry and after 24 h or 1 week in water. Samples (*n *= 3) were equilibrated for 1 min under 1 mN load at room temperature. They were subsequently subjected to a constant load of 500 mN for 10 min. This load was then rapidly reduced and held at 1 mN for 5 min to assess strain relaxation (recovery). Per cent strain was recorded as a function of time at room temperature.

#### 2.2.3 Wettability and surface free energy

The surface wettability and free energy of the polymer and all composites were determined (*n *= 5) by measuring the contact angle of water (polar liquid) and diiodomethane (non-polar liquid) droplets (∼2.5 μl) on the surface of each specimen using a KSV200 (KSV instruments, Finland). The drop profile was recorded at 1 s intervals for 1 min. The calculation of surface free energy was carried out using the OWRK method via KSV software [31]. Studies were undertaken with dry specimens (stored at room temperature in sealed plastic bags) and others stored in water for 24 h and 1 week.

#### 2.2.4 Human mesenchymal stem cell attachment and proliferation

For cell compatibility investigations, four discs of each formulation were snugly fitted into 48 well plates. These discs were 300 μm thick and 10 mm in diameter. Plates were then sterilized with UV light, and pre-treated with 1 ml of growth medium (DMEM, 10% foetal calf serum, and 1% penicillin and streptomycin (Gibco)). After 24 h in a 37 °C incubator with 5% CO_2_ in air, this medium was replaced with fresh medium providing 3 × 10^4^ human mesenchymal stem cells per cm^2^.

For a qualitative study of cell attachment, at 4 and 24 h, cells were fixed overnight with 3% glutaraldehyde in 0.1 M sodium cacodylate buffer (Agar Scientific Ltd., Essex, UK) at 4 °C, and then dehydrated in a series of graded alcohols. The dehydrated samples were further dried in hexamethyldisilazane (HMDS, Taab Laboratories Ltd., Berkshire, UK) for 1 min, and then left to air dry. The fully dried samples were finally mounted on aluminium stubs, sputter coated with gold–palladium alloy, and viewed by scanning electron microscopy (JEOL JSM 5410LV, USA). One disc of each formulation was used at each time point.

Cell proliferation was quantified using the remaining three samples of each formulation and an Alamar Blue assay®. After 1, 3 and 7 days, the growth medium of was replaced by 10% v/v Alamar Blue® and the cultured samples were incubated for 4 h according to the manufacturer’s instructions. Fluorescence of the subsequently removed medium was measured using a Fluroskan Ascent plate reader (Labsystems, Helsinki, Finland) and corrected for background.

## 3 Results

Incorporation of MCPM/β-TCP filler increased the viscosity of the formulations but even with 70 wt.% filler addition the formulations still flowed. Visual inspection of the set materials showed no evidence of uncured surface monomer with either the polymer or composites as indicated by loss of shine as the set material has a dull appearance.

### 3.1 Water sorption and degradation in growth medium

**Fig. 1:**
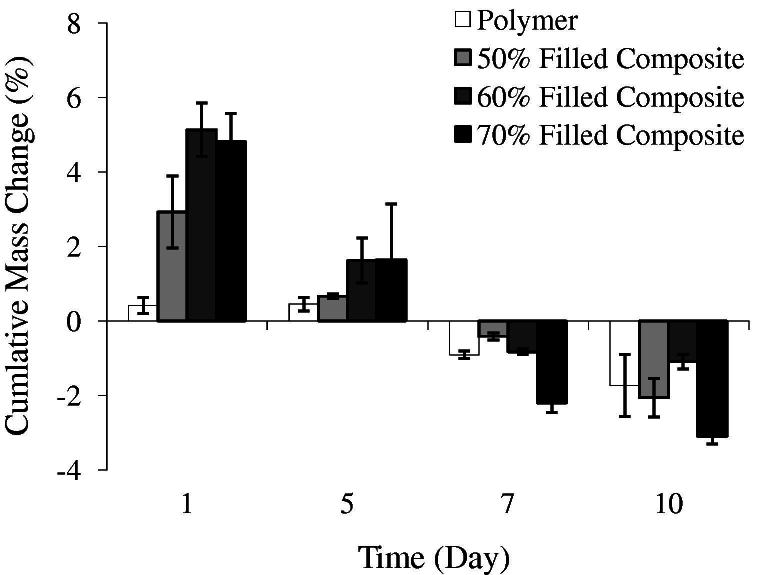
Percentage mass changes, in standard growth medium at 37 °C, of composites filled with 50–70 wt.% calcium phosphate filler and unfilled polymer. *n* = 3; error bars are ±SD.

An initial rise in wet mass, as a result of early water sorption and polymer expansion, was observed for all cured formulations. From 24 h, wet mass declined linearly with time due to degradation. Early water sorption and subsequent mass loss rate (estimated from the intercept and gradient of wet mass vs. time) increased nearly 6- and 4-fold, respectively, with 70 wt.% filler inclusion (see Fig. 1 and Table 1).

### 3.2 Mechanical properties

#### 3.2.1 Dynamic mechanical thermal analysis

**Fig. 2:**
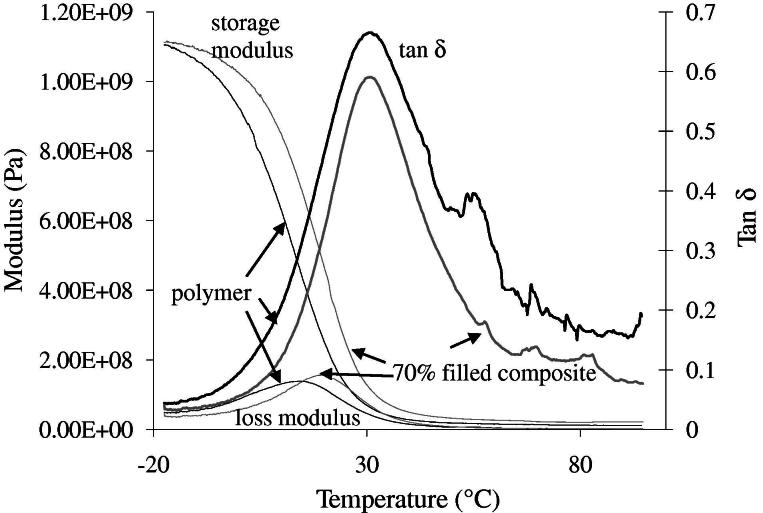
Example plots of storage modulus ($E^{\prime }$), loss modulus ($E^{\prime \prime }$) and tan *δ* vs. temperature for PPGLDMA filled with 0 or 70 wt.% MCPM/β-TCP.

Examples of modulus and tan *δ* vs. temperature are provided in Fig. 2. Increasing filler loading caused no obvious changes in tan *δ* or *E*′ and *E*″ at low and high temperatures (data not shown). The position of rapid *E*′ decline and the *E*″ peak, however, was at significantly higher temperature for the composites compared with the polymer. The glass transition temperatures estimated from the *E*″ peak position are given in Table 1.

#### 3.2.2 Quasi-static test

**Fig. 3:**
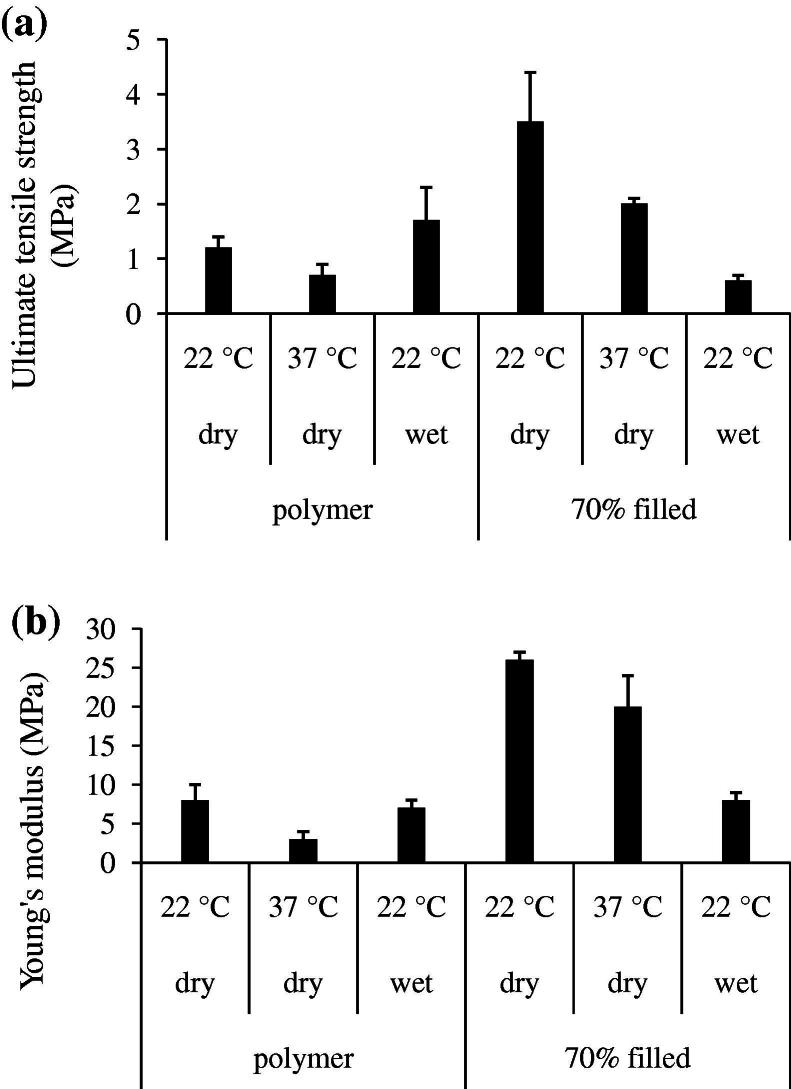
Average ultimate tensile strength (a) and Young’s modulus (b) of PPGLDMA filled with 0 and 70 wt.% MCPM/β-TCP. Specimens had either been kept dry or stored for 24 h in water. Studies were performed at 22 °C. Additionally, with dry samples, measurements were made at 37 °C. *n = *5; error bars are ±SD.

Increasing MCPM/β-TCP filler addition raised initial, room-temperature, quasi-static polymer strength and modulus by up to 3-fold but reduced per cent strain at break (Table 1). Raising the temperature to 37 °C reduced strength and modulus of both polymer and composite (Fig. 3). Twenty-four hours in water caused an even greater decline in composite strength and modulus but had negligible effect on the polymer (Fig. 3).

#### 3.2.3 Creep/recovery experiment

**Fig. 4:**
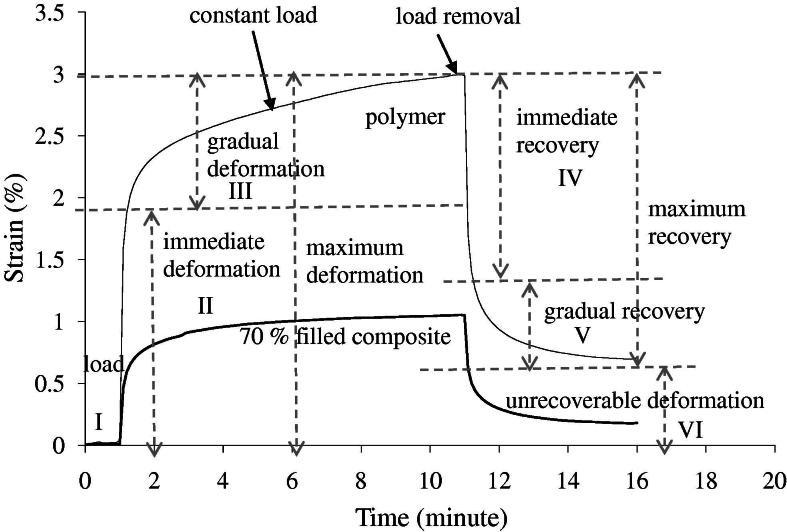
Examples of single specimen deformation behaviour on application of 500 mN load and recovery for the dry polymer and 70 wt.% filled composite.

Creep/recovery for the dry polymer and 70 wt.% filled composite is shown in Fig. 4. Several regions can be identified. Regions I–III represent equilibration time and immediate and subsequent creep deformation periods in order. These are followed by immediate and then slow (viscoelastic) recovery periods. Average strain and recovery percentages for the dry polymer and 70 wt.% filled composite are given in Table 2. Immediate, gradual and unrecoverable strains were all 4- to 6-fold higher for the polymer than the composite. After 24 h (data not shown) or 1 week in water (Table 2), creep recovery results for the polymer were not significantly altered. After 24 h in water, however, the application of a 500 mN load caused the composite samples to break.

### 3.3 Wettability and surface free energy

The water contact angle for different formulations was 70 ± 5° while the diiodomethane contact angle was 35 ± 3°. Filler addition slightly reduced the total and dispersive surface energy but its effect on the polar surface energy was not experimentally significant (see Table 3). Storage in water for 24 h or 1 week had no significant effect on any surface energy values (data not shown).

### 3.4 Human mesenchymal stem cell attachment and proliferation

**Fig. 5:**
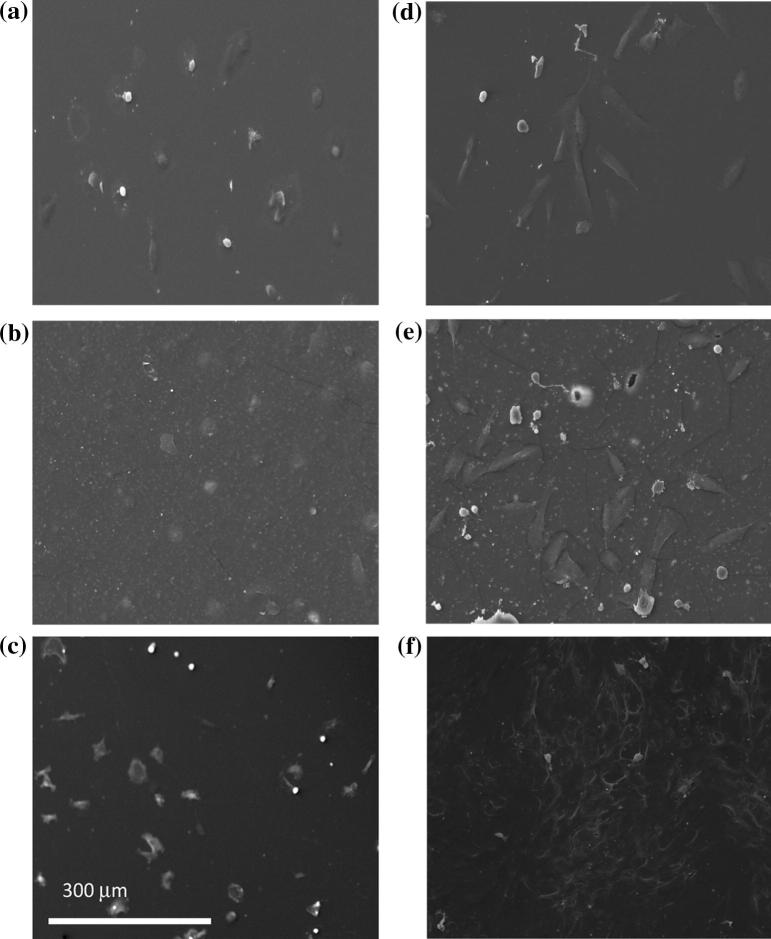
SEM images showing early attachment of human mesenchymal stem cells seeded on the surface of 300 μm thick sheet of the polymer (a and d), composites filled with 60 wt.% MCPM/β-TCP filler (b and e), and Thermanox® (cf. positive control surface) after 4 h (a–c) and 24 h (d–f) of culture.

Fig. 5a–c shows SEM images of random areas of representative samples after 4 h of culture. At this time the human mesenchymal cells had attached to all surfaces and were rounded or spindle shaped, which is typical for early attaching cells. At 24 h, fibroblast-like morphology became more evident due to greater cell spreading and attachment (Fig. 5d–f). Cells on the positive Thermanox® control, however, appeared more flattened and to occupy a larger percentage of the surface.

**Fig. 6:**
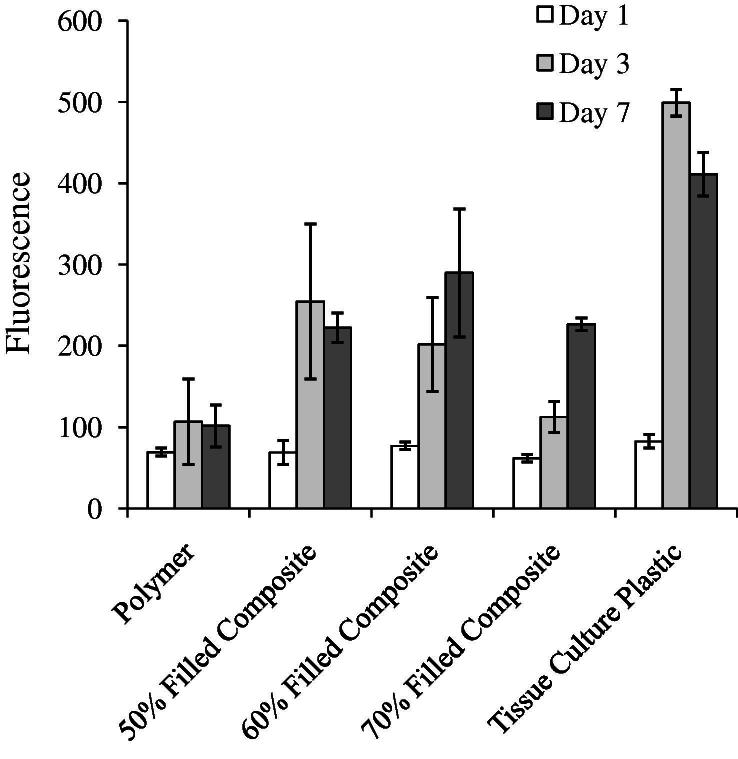
Background-subtracted Alamar® blue fluorescence due to active human mesenchymal stem cells growing on the surface of composites filled with 50–70 wt.% MCPM/β-TCP, polymer or tissue culture plastic control at 1, 3 and 7 days. Bars represent average values (*n *= 3); error bars are ±SD.

In cell proliferation studies, at day 1 of culture, there was a slightly lower number of cells growing on the polymer and different composite surfaces compared with the positive control (tissue culture plastic). From day 3, cell numbers on average increased significantly in the order polymer < composites < control (Fig. 6).

## 4 Discussion

### 4.1 Water sorption and degradation in growth medium

The degradation kinetics of PPGLDMA adhesives have a complex dependence upon polymer structure [13,32], filler type/content [17–19] sample thickness [15] and time. Typically, however, these materials exhibit fast initial water sorption that substantially slows after the first 24 h. This process may enhance bulk polymer hydrolysis that subsequently enables faster surface erosion. After 24 h, the wet mass declines due to a combination of bulk filler replacement by lower density water and surface erosion.

Comparison of the above new work with earlier studies using thicker samples of the same composition [18] indicates that the early percentage water sorption is lower for thinner polymer and composite samples. This is contrary to what is expected by Fick’s law. Percentage wet mass decline, however, is barely affected by thickness. There is therefore slower mass loss with thinner samples. Lower mass loss and water sorption with thin samples is anomalous but has been observed in one other study with PPGLDMA composite compositions [15]. This was shown to be a consequence of reduced bulk polymer hydrolysis in thinner samples. This may be a result of reduced acid-catalysed changes as acidic protons may be released more rapidly from thinner specimens.

The above new work confirms that MCPM addition enhances both early water sorption and wet mass loss. MCPM near the surface of the materials can dissolve; MCPM in the bulk reacts with β-TCP to form less-soluble dicalcium phosphates. This means that the chemistry of thin samples is quite different from that of thick specimens.

### 4.2 Mechanical properties

#### 4.2.1 Dynamic mechanical thermal analysis

The storage modulus results indicate that at just a few degrees below room temperature the dry materials all behave like rigid, high-modulus plastics but at body temperature are crosslinked rubbers. A random copolymer glass transition temperature is typically between that estimated using Eqs. (1) and (2) [33]:

(1) $$1/T_{g}=\sum w_{i}/T_{g\text{,}i}$$

(2) $$T_{g}=\sum w_{i}T_{g\text{,}i}$$

where *w_i_* and *T_g,i_* are the weight fractions and glass transition temperatures of the pure polymer components. Assuming glass transition temperatures of 203 [34], 333 [34] and 380 K [35] for polypropylene glycol, polylactide and polymethylmethacrylate, respectively, the *T_g_* of PPGLDMA is predicted to be 277 K (4 °C) and 298 K (25 °C). The small increase in *T_g_* with filler addition suggests there may be some weak interaction between the polylactide ester groups [36] and filler further immobilizing the rubber matrix phase [30]. The level of change, however, is not sufficient to change the material from a rubber to glass at body temperature. Having the PPGLDMA glass transition temperature just below body temperature may be beneficial because the loss modulus is then close to its maximum value. The material is then capable of absorbing higher levels of stress [37].

At room and body temperatures, storage material modulus values were higher, as expected, than their corresponding Young’s moduli [38]. Low-modulus/rubber-like behaviour is beneficial as it will enable micromotion but not excessive movement in a bonded material–bone interface. Micromotion up to between 50 and 150 μm can aid bone ingrowth. Beyond this level, however, excessive movement can lead to unwanted fibrous tissue formation [39].

A further benefit of having a low *T_g_* is that polymerization can be more complete. As a polymerization process continues, the growing polymer *T_g_* increases. The reaction, however, can halt when the *T_g_* reaches that of the surroundings and the polymer changes from rubber to plastic. In the case of PMMA this may lead to unwanted toxic monomer remaining within the set cement [40]. Previous work with PPGLDMA has shown that >85% conversion is typical in both polymer and composites [18]. With dimethacrylates such as PPGLDMA there can potentially be no free monomer with just 50% conversion. Theoretical studies on dimethacrylates indicate that with 85% reaction, unbounded monomer content should be very low [41].

#### 4.2.2 Quasi-static test

The strength of the PPGLDMA polymer is two orders of magnitude lower than that of PMMA [42] or cortical bone [43], although comparable with that of cancellous bone [44]. Addition of the filler particles could raise the initial strength but this advantage was soon lost upon water immersion. The more significant reduction in composite strength with just 24 h water immersion was presumably due to higher water sorption and bulk degradation. These results suggest the materials might be of use in low-load-bearing bone but in high-load situations would need to be used in conjunction with other support devices such as more conventional polylactide pins and screws.

The Young’s modulus of the PPGLDMA polymer at both room and body temperature were of similar magnitude to that of new bone consisting primarily of collagen and water but several orders of magnitude lower than bone fully mineralized with hydroxyapatite. Considering the orders of magnitude differences in polymer and filler modulus, the effect of high filler addition had only a minor effect on the Young’s modulus of PPGLDMA. This was probably due to limited interaction between the flexible matrix and rigid filler phases preventing the later from contributing to this property. Given their low modulus, the polymer and composites might be of use as thin flexible adhesive layers to aid early bone–bone or bone–metal bonding. Alternatively they might be injected within osteoporotic bone to act as a stress absorber and provide additional anchorage for screws.

If strength is divided by modulus, average values of 20% and 10% strain for the polymer and composite, respectively, are obtained. The similarities between these values and strain at break observed in Table 1 indicate that the stress–strain curve is mostly linear with limited plastic deformation after the elastic region. In the elastic region the polymer chains are reversibly stretched. The lack of significant plastic deformation is a result of the high level of crosslinking within the materials, preventing more permanent whole-chain polymer movement [45].

#### 4.2.3 Creep/recovery experiment

The viscoelastic behaviour recorded for the experimental adhesives can be described by a Voight–Maxwell model [46]. The immediate reversible strain could be a result of polymer chain stretching. The gradual and irreversible strain could be due to chain disentanglement. In the composites there are fewer chains to stretch and disentangle, causing the reduction in strain percentages. The greater change in properties for the composite upon water immersion is consistent with greater bulk degradation.

### 4.3 Wettability and surface free energy

In this study, the recorded water contact angle for the PPGLDMA (70 ± 5°) is comparable with that reported for both PLA-A [47] and PMMA [48]. The dispersive free energy is also comparable with that for PMMA (36 units) and other common polymers with hydrocarbon chains but slightly higher than that of PPG (31 U). The polar free energy of PPGLDMA is greater than that of PMMA (6) or PPG (0.4), but about half that of hydrophilic poly(HEMA) (20) [49]. It is possible that the presence of small amounts (10 wt.%) of poly(HEMA) in the PPGLDMA formulations causes the rise in the polar free energy. The lack of change in this energy upon water immersion suggests that the production of acid groups upon degradation of the polymer has a limited effect upon this property. The presence of the filler reduces both dispersive and polar free energies. High surface free energy can increase wetting on surfaces but will also reduce capillary action and the penetration of an adhesive into pores. A balance between these two properties is required for adhesives.

### 4.4 Human mesenchymal stem cell attachment and proliferation

In previous studies [18], human mesenchymal cells were unable to attach to 2 mm thick samples after preconditioning in growth medium for 24 h. This problem was overcome through further preincubation in the presence of foetal calf serum for an additional 24 h. The above new study has shown this problem can also be overcome by use of thinner samples. This is presumably due to a reduction in the levels of acidic degradation products with thinner samples. These are mostly released into the surrounding medium within the first 24 h [18,19].

Filled adhesive formulations supported faster proliferation (observed as higher metabolic activity) than the polymer. This could be due to the previously observed neutralization of the acidic polymer degradation products by calcium phosphate filler [17–19] and/or interaction between the cells and calcium.

## 5 Conclusions

Thinner samples of PPGLDMA exhibit reduced water sorption and associated bulk degradation. Even with 70 wt.% filler addition, the PPGLDMA composites behave like flexible crosslinked rubbers at body temperature. Filler addition increases the initial strength and Young’s modulus. This effect is, however, lost upon water immersion due to the increased water sorption and degradation upon filler addition. The Young’s modulus was less affected by increasing the temperature from room to body temperature. With dry samples, filler addition reduced both reversible and irreversible deformation under load. The PPGLDMA formulations may have higher polar surface free energy than PMMA due to the inclusion of 10 wt.% poly(HEMA). This might enable the degradable formulations to more readily spread upon and subsequently bond to bone. Use of thinner samples and addition of reactive filler improved cell proliferation on PPGLDMA.

## Figures and Tables

**Table 1:** Average intercept and gradient of percentage mass change vs. time (related to early water sorption and degradation rate, respectively), glass transition temperature and static mechanical properties, of PPGLDMA filled with 0, 50, 60 or 70 wt.% MCPM/β-TCP. *n* is sample number; errors are ±SD.

Materials	Mass loss (*n *= 3)		DMTA (*n *= 3)	Initial static mechanical properties (22 °C) (*n *= 5)		
Intercept (%)	Gradient (%/day)	*T_g_* (°C)	Strain at break (%)	Yield strength (MPa)	Young’s modulus (MPa)
Polymer	1.0 ± 0.3	0.3 ± 0.1	14 ± 1	20 ± 3	1.2 ± 0.2	8 ± 2
50 wt.% filled composite	3.5 ± 0.9	0.6 ± 0.1	22 ± 2	14 ± 2	1.7 ± 0.3	17 ± 2
60 wt.% filled composite	5.4 ± 0.6	0.7 ± 0.1	20 ± 2	12 ± 1	2 ± 0.2	20 ± 2
70 wt.% filled composite	5.7 ± 0.6	0.9 ± 0.1	19 ± 1	12 ± 2	3.5 ± 0.9	26 ± 1

**Table 2:** Parameters from creep/recovery experiments recorded for 70 wt.% composite and polymer (dry and after 1 week water immersion at 22 °C). The results are average values ± SD (*n *= 3). No results for composite at 1 week are given as the samples broke.

Parameter of creep/recovery experiment	Polymer		70 wt.% filled composite dry at 22 °C
Dry at 22 °C	1 week water immersion at 22 °C
Immediate strain%	2.0 ± 0.5	2.2 ± 0.1	0.5 ± 0.0
Maximum gradual strain%	3.9 ± 0.8	3.6 ± 0.1	0.9 ± 0.1
Gradual strain%	1.9 ± 0.6	1.4 ± 0.1	0.5 ± 0.1
Immediate recovery%	1.3 ± 0.3	1.1 ± 0.1	0.4 ± 0.1
Gradual recovery%	1.0 ± 0.2	0.7 ± 0.1	0.3 ± 0.1
Unrecoverable strain%	1.1 ± 0.5	1.8 ± 0.5	0.2 ± 0.1

**Table 3:** Surface free energy (mN min^−1^) of composites filled with 50–70 wt.% MCPM/β-TCP and polymer. The results are average values ± SD (*n *= 5).

Materials	Surface free energy (mN min^−1^)		
SFE^d^	SFE^P^	SFE^tot^
Polymer	37 ± 1	11 ± 2	48 ± 3
50 wt.% filled composite	35 ± 2	8 ± 1	42 ± 2
60 wt.% filled composite	33 ± 2	10 ± 2	43 ± 2
70 wt.% filled composite	34 ± 1	7 ± 3	41 ± 2
